# Functional Analysis of Sugars in Modulating Bacterial Communities and Metabolomics Profiles of *Medicago sativa* Silage

**DOI:** 10.3389/fmicb.2020.00641

**Published:** 2020-05-05

**Authors:** Bing Wang, Run Gao, Zhe Wu, Zhu Yu

**Affiliations:** ^1^State Key Laboratory of Animal Nutrition, College of Animal Science and Technology, China Agricultural University, Beijing, China; ^2^College of Grass Science and Technology, China Agricultural University, Beijing, China

**Keywords:** alfalfa, metabolomics, microbial community, pectin, triterpenes aglycones

## Abstract

This study explored the effects of four sugar source treatments, including no sugar (CON), fructose (FRU), pectin (PEC), and starch (STA), on the microbiota and metabolome of alfalfa (*Medicago sativa*) silage. The bacterial community was determined via 16S rRNA gene sequencing. The metabolome was analyzed using ultra high-performance liquid chromatography tandem time-of-flight mass spectrometry (UHPLC/TOF-MS). After 60 days of fermentation, the pH values in FRU and PEC were lower than those in STA and CON. FRU had a greater lactic acid concentration compared to STA and CON. *Weissella* (47.44%) and *Lactobacillus* (42.13%) were the dominant species in all four groups. The abundance of *Pediococcus* was lower, and the abundance of *Leuconostoc*, *Pantoea*, and *Microbacterium* was higher, in FRU compared to CON. The abundance of *norank_f__Bacteroidales_S24-7_group* was higher, and the abundance of *Turicibacter* was lower, in both FRU and PEC than in CON. *Leuconostoc* was negatively correlated with the pH value, and *Pediococcus* was positively correlated with the pH value. No microbiomes were detected as discriminative features between STA and CON. The addition of FRU and PEC presented more peptides, such as Leu–Val–Thr, Leu–Phe, Ile–Pro–Ile, Val–Trp, and Ile–Leu–Leu but a lower abundance of metabolites for triterpene glycosides including sanchinoside B1, medicagenic acid, betavulgaroside IV, and prosapogenin compared to CON. The addition of PEC presented more phenyllactic acid compared to CON. Our study demonstrated that the addition of pectin and fructose improved the quality of alfalfa silage mainly by promoting *Leuconostoc*, *Pantoea*, and *Microbacterium*, and inhibiting *Pediococcus* in FRU, and promoting *norank_f__Bacteroidales_S24-7_group* and inhibiting *Turicibacter* in both FRU and PEC; this was due to altered metabolic profiles resulting from antifungal activity and decreased triterpene glycoside accumulation. This study improves our understanding of ensiling mechanisms related to the contributions of sugar.

## Introduction

Alfalfa (*Medicago sativa* L.) is one of the most important forage crops as animal feed and is commonly used for ensiling worldwide (Dunière et al., 2013). Silage is an ideal method for maintaining forage because it can prolong the stable storage period of forage and help overcome the discrepancy between livestock production and the seasonal imbalance of available high-quality forage (Wright et al., 2000). However, compared to other forages, it is difficult to make silage with alfalfa because of its high buffering capacity, high crude protein (CP) level, and low water-soluble carbohydrate (WSC) content (McDonald et al., 1991). As one of the main roughages in dairy cow production, alfalfa silage can provide high-quality nutrients for livestock production. Generally, the addition of sugar sources such as molasses and fructose, added individually or mixed with other feed ingredients rich in sugar, is an effective way to increase the WSC content of alfalfa (Li et al., 2018; Muck et al., 2018; Zhang et al., 2018). It was found that pectin can be easily used by ruminants (Mertens, 2002; Liu et al., 2015), and pectin was classed as soluble and easily fermented carbohydrates based on the Cornell Net Carbohydrate and Protein System model (Sniffen et al., 1992). The alfalfa showed the beneficial function of improved rumen fermentation in dairy cows partly due to its abundant pectin, an easily fermented carbohydrate (Wang et al., 2014). Thus, the addition of exogenous pectin may have efficient roles in silage fermentation by lactic acid bacteria (LAB) due to its easily fermentable properties. Starch is also an important easily fermented carbohydrate for ruminants, which is an important factor for determining the quality of corn silage and its effect on milk synthesis (Ferraretto et al., 2018). To our knowledge, however, few studies have analyzed the effects of the addition of pectin or starch to see their effects on alfalfa silage fermentation.

Epiphytic microbial communities or inoculants play critical roles in ensiling forage during fermentation, which produces various metabolites and involves many types of microorganisms (Kung et al., 2018). Several studies have analyzed silage microbiome and metabolome profiles to help understand the biological process underlying silage formation using integrative 16S rRNA sequencing and metabolomics (Guo et al., 2018; Xu et al., 2019). Metabolomics can help offer more information, including metabolic processes and indicators of silage quality based on the amino acid, fatty acid, oligosaccharide, vitamin, peptide, flavoring agent, and aromatic compound contents, but not conventional organic acids, including lactic acid, acetate, propionate, and butyrate (Sun et al., 2012). Therefore, the unknown metabolites, newly functional LAB, and the consequential metabolic process during ensiling could be revealed by an integrative analysis of the metabolome and microbiome.

To our knowledge, no previous work has performed LC–MS to identify the metabolome of alfalfa silage. We hypothesize that different roles and correspondingly various mechanisms exist, underlying the ensiling quality between sugar sources, especially for pectin. Thus, the current study aimed to detect the effects of different sugar sources (easily fermented carbohydrates) on silage quality and the underlying mechanism related to the contribution of sugars to the fermentation of alfalfa silage by integrative 16S rRNA sequencing and metabolomics.

## Materials and Methods

### Experimental Design

The experiment was conducted in October 2018 at the China Agriculture University Farm (Yanchi, Ningxia Hui Autonomous Region). Alfalfa was planted in fields of loam soil, and no herbicides or fertilizers were applied. Alfalfa was harvested at the squaring stage after the third cutting. After wilting to approximately 35% dry matter basis, the alfalfa was chopped into 1–2-cm segments with a forage cutter (Lingong Machinery, Shandong, China). The chopped forage was sampled and analyzed for chemical composition. The concentrations of CP, neutral detergent fiber (NDF), acid detergent fiber (ADF), and WSC in alfalfa before ensiling were 19.4, 37.8, 28.5, and 6.29% of the dry matter basis, respectively (Supplementary Table S1). The fermentation characteristics, including pH value and organic acid concentration, were also detected (Supplementary Table S1). Four sugar treatments including a control (CON), fructose (FRU, d -fructose, ≥ 99% purity), pectin (PEC, 65% purity, from citrus), and starch (STA, corn starch, 86.6% dry matter basis) were used for silage and evaluated with three replicates each. The addition of fructose can be treated as a positive control. The dose of each sugar that was added to the fresh forage was 2% of the fresh matter of alfalfa before ensiling. For all treatments, 1 × 10^6^ cfu/g of fresh matter *Lactobacillus plantarum* was added as described previously (Zhang et al., 2015). The mixed material (300 g) was packed into polyethylene oxygen isolation plastic film bags (Hiryu KN type; dimensions: 180 mm × 260 mm; thickness: 0.2 mm; embossed foodsaver bag; Asahikasei, Tokyo, Japan). Then the bags were sealed with a vacuum sealer (BH950; Matsushita, Tokyo, Japan) and stored at ambient temperature (22–25°C) in dark conditions for 60 days of ensiling.

### Sample Collection and Measurements

The samples were dried in a forced-air oven at 65°C for 48 h, and bags were opened to obtain the dry matter content. The air-dried samples were ground through a 1-mm screen using a mill (FZ102, Test Instrument, Tianjin, China) and stored for further chemical analysis of CP (method 988.05; AOAC, 1990). The concentrations of NDF and ADF were determined using thermostable α-amylase and sodium sulfite in an Ankom Fiber Analyzer (A2000I; Ankom Technology, Macedon, NY, United States) following the procedure of Van Soest et al. (1991). The results were expressed on a dry matter basis without residual ash.

Twenty grams of each silage sample was mixed with 180 ml of distilled water, homogenized in a blender for 30 s, and filtered through four layers of cheesecloth and filter paper successively (Tian et al., 2017). The pH was measured immediately using a pH meter (PHS-3C; INESA Scientific Instrument, Shanghai, China). The concentrations of lactic acid, acetate, propionate, and butyrate were measured by high-performance liquid chromatography (HPLC; Shimadzu, Tokyo, Japan) (Xu et al., 2007). The contents of ammonia–nitrogen (AN) were determined according to Broderick and Kang (1980), and the ammonia N was calculated and expressed as % of total N (AN/TN).

### DNA Extraction and Sequencing

The microbial pellet was obtained from silage according to the procedure from a previous study (Zheng et al., 2017). The 16S rRNA V3–V4 region of the eukaryotic ribosomal RNA gene was amplified using the following primers: 338F, ACTCCTACGGGAGGCAGCAG and 806R, GGACTACHVGGGTWTCTAAT, where the barcode was an eight-base sequence that was unique to each sample. The detailed PCR procedure was according to our previous study (Sun et al., 2019). Purified amplicons were pooled in equimolar amounts and paired-end sequenced (2 × 300) on an Illumina MiSeq PE300 platform (Illumina, San Diego, CA, United States) according to the standard protocols by Majorbio Bio-Pharm Technology Co. Ltd. (Shanghai, China). In brief, (1) “Y” adapters were linked; (2) adapter dimers were removed using beads; (3) PCR amplifications were conducted for all library conditions; and (4) single-stranded DNA fragments were generated using sodium hydroxide. The raw reads were deposited into the NCBI Sequence Read Archive (SRA^1^) database (Accession Number: SRP197138).

### Bioinformatic Analysis

Raw fastq files were quality filtered by Trimmomatic and merged by FLASH with the criteria (Sun et al., 2019). Then, the filtered reads were used for pairing, and the merged reads were used for operational taxonomic unit (OTU) clustering, taxonomic classification, and community diversity assessment. OTUs were clustered with ≥97% similarity cutoff using UPARSE (version 7.1^2^) with a novel “greedy” algorithm that performs chimera filtering and OTU clustering simultaneously (Edgar, 2013). Between groups, a Venn analysis was constructed to identify unique and common OTUs. The taxonomic affiliation of each 16S rRNA gene sequence was analyzed by RDP Classifier algorithm^3^ (Version 2.2) (Wang et al., 2007) based on the SILVA (Pruesse et al., 2007) database^4^ with a confidence threshold of 70%. At the beginning of data analysis, data normalization was carried out for all samples, and subsequent alpha diversity and beta diversity were based on normalized data. The OTU rarefaction curves were plotted in QIIME. In addition, alpha diversity and beta diversity indices were calculated in QIIME. To compare the alpha indices among groups, Tukey’s HSD test and a Kruskal–Wallis H test were performed. For comparisons of the four groups, the Bray–Curtis dissimilarity metric and permutational multivariate analysis of variance (PERMANOVA) were performed using the vegan package in the R programming environment (Oksanen et al., 2007; Berg et al., 2016). Bray–Curtis distance matrices were constructed using rarefied OTU abundance and visualized by principal coordinate analysis (PCoA).

### LC-MS Analysis

Samples of ensiled alfalfa forages (5 g) were extracted with 20 ml of cold extraction liquid (methanol: water = 4:1, vol:vol) in EP tubes. The LC-MS analyses were performed using an AB SCIEX’s UPLC–TripleTOF system with ultra HPLC tandem time-of-flight mass spectrometry (UHPLC/TOF-MS). The detailed information of the procedure of UHPLC/TOF-MS was according to our previous study (Wang et al., 2019). To evaluate the stability of the analytical system during the experiment, a quality control (QC) sample was prepared every six samples.

### Metabolomic Data Preprocessing

The MS raw data files were converted for pre-processing with the software Progenesis QI (Waters Corporation, Milford, MA, United States) before statistical analysis was performed. The preprocessing results generated a data matrix that consisted of the baseline filtration, peak identification, integration, retention time correction, peak alignment, and finally a data matrix of retention time, mass-to-charge ratio, and peak intensity. Variables with non-zero values above 50% in all samples were retained; the missing values with one-half of the minimum value in the original matrix were filled; the total peaks were normalized, and the QC samples with relative standard deviation (RSD) ≥ 30% were deleted. It was concluded that the LC/MS analytical system had appropriate stability and repeatability and that the acquired data were of good enough quality for subsequent assays.

### Multivariate Statistical Analysis and Identification of Metabolites

The metabolite annotation and data pretreatment were conducted using Progenesis QI (Waters Corporation, Milford, MA, United States) software, combined with commercial databases, including KEGG^5^ and HMDB^6^. The peaks were detected, and the metabolites were identified through the interquartile range denoising method. Missing raw data values were set at half of the minimum value of the detection limit. In addition, an internal standard normalization method was employed in these data analyses. The principal component analysis (PCA), partial least-square discriminant analysis (PLS-DA), and orthogonal projections to latent structure discriminant analysis (OPLS-DA) were analyzed by the ropls (Version 1.6.2) software package. The differential metabolites (DMs) were detected based on the OPLS-DA analysis. The first principal component of variable importance in the projection (VIP) was obtained. The VIP values exceeding 1.1 were first selected as changed metabolites. Then, the remaining variables were assessed by Student’s *t*-test (*P* < 0.01). The fold-change (FC) value of each metabolite was calculated by comparing the mean values of the peak area obtained from any comparison, and the log_2_FC value was used to indicate the specific variable quantity in the comparison. The false discovery rate (FDR) of each metabolite was computed from the measurements using the fdrtool package in R (Version 3.2.4). The DMs were identified based on VIP > 1.1, *P* < 0.01, |log_2_FC| > 0.8, and FDR < 0.01. In addition, MetaboAnalyst 4.0, a web-based tool for the visualization of metabolomics^7^, was utilized to generate a heatmap, which was used to visualize the relative abundance of DMs in each sample (Chong et al., 2018).

### Correlations Analysis

A Spearman’s rank correlation matrix was generated by calculating Spearman’s correlation coefficient to explore the correlations. The correlations between fermentation characteristics and the top 10 bacterial genera and species were visualized using the Pheatmap6 package in R (Version 3.2.4), with values of *P* < 0.05 and |*r*| ≥ 0.59 as significant. The functional correlations between the different bacteria and DMs were visualized using Cytoscape 3.6.1 (Smoot et al., 2011), with *P* < 0.01 and |*r*| ≥ 0.94 as significant.

### Statistical Analysis

The data of microbiota and metabolome were analyzed on the free online platform Majorbio I-Sanger Cloud Platform^8^. The fermentation and nutritional characteristics of alfalfa silage were analyzed using one-way analysis of variance based on the general linear model (GLM) procedure of SAS (version 9.2; SAS Institute Inc., Cary, NC, United States). LEfSe discriminant analysis was utilized to select and demonstrate the differentially abundant taxa between groups (LDA score ≥ 2.5). The Mann–Whitney *U*-test was used to examine the differential bacterial communities between two groups at the genus level. Significance was declared at *P* < 0.05.

## Results

### Silage Fermentation and Nutritional Characteristics

The pH values of FRU and PEC were both lower compared to STA and CON (*P* < 0.01) (Table 1). The lactic acid concentration was higher in FRU compared to CON (*P* < 0.01) and STA (*P* = 0.02). The ADF concentration was lower in FRU (*P* < 0.01), PEC (*P* = 0.02), and STA (*P* = 0.01) compared to CON. No butyrate was detected among the four groups.

**TABLE 1:** Chemical compositions and fermentation characteristics of alfalfa silage prepared with different sugars sources.

Items^*b*^	Treatments^*a*^				SEM	*P*-value
	CON	FRU	PEC	STA		
pH	4.85^*a*^	4.33^*b*^	4.41^*b*^	4.75^*a*^	0.042	<0.01
AN/TN,%	4.79	4.83	4.62	5.25	0.260	0.28
Lactic acid, g/kg DM	36.9^*b*^	74.6^*a*^	53.9^*ab*^	44.2^*b*^	8.92	0.03
Acetate, g/kg DM	15.6	24.0	17.5	19.2	4.02	0.37
Propionate, g/kg DM	1.19	1.96	1.36	1.55	0.288	0.19
Butyrate, g/kg DM	ND	ND	ND	ND		
CP, g/kg DM	194.9	193.5	197.0	192.0	2.22	0.32
NDF, g/kg DM	396.9	381.0	387.2	377.9	10.33	0.54
ADF, g/kg DM	308.2^*a*^	287.7^*b*^	292.0^*b*^	288.9^*b*^	4.31	0.03

*^a^CON, control; FRU, fructose; PEC, pectin; STA, starch. ^b^DM, dry matter; AN/TN, ammonia nitrogen/total nitrogen; ND, not detected.*

### Bacterial and Metabolomic Profiles in the Silage

**FIGURE 1:**
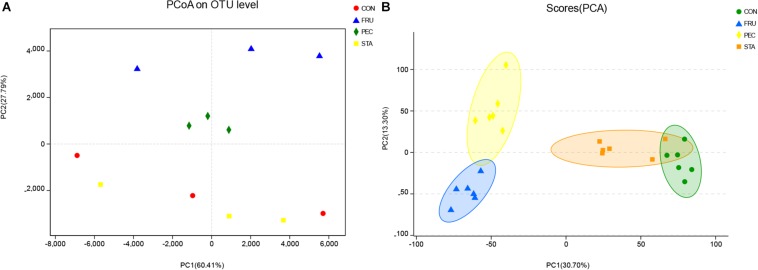
The principal coordinate analysis (PCoA) plot **(A)** showing variation in bacterial community structure (PERMANOVA test with 999 permutations, *P* = 0.03) (see Supplementary Table S2) and the principal component analysis (PCA) of metabolites **(B)** showing variation among the four alfalfa silage treatments with no sugar (CON), fructose (FRU), pectin (PEC), and starch (STA) after 60 days of ensiling. Each point represents an individual sample.

In total, 1,193,852 raw reads were obtained for the bacterial 16S rRNA genes in the four groups. After removing short and low-quality reads, singletons, triplicates, and chimeras, the number of sequences (total and average) was 596,926 and 49,744, respectively. The number of sequences (total and average) after filtering and subsampling was 462,972 and 38,639, respectively. The Good’s coverage value for all samples was greater than 99.9%. The rarefaction curves showed clear asymptotes, and the number of reads was the same for all samples after normalization (Supplementary Figure S1). A total of 105 OTUs, ranging from 72 to 84 per sample, were identified. There were 96, 96, 95, and 98 OTUs identified in CON, STA, FRU, and PEC, respectively, and 81 OTUs were found in all four groups, which accounted for 77.1% of the total OTUs (Supplementary Figure S2). No differences in OTUs were found among the four treatments. No differences in the indices of Sobs, Shannon, Simpson, Ace, Chao, and coverage analysis were found among the four groups (Table 2). The PCoA results with Bray–Curtis distances at the OTU level indicated that FRU and PEC were separated from CON, but STA was not (Figure 1A and Supplementary Table S2). For the metabolomic profiles, no obvious separation was found between STA and CON according to the PCA plot (Figure 1B). Good separation between FRU and CON and between PEC and CON was achieved, as shown in the PCA score plots (Figure 1B). The parameters for the classifications, including PLS-DA and OPLS-DA, determined by software were stable and relevant to fitness and predictions (Supplementary Table S3).

**TABLE 2:** Diversity statistics of bacterial community during ensiling.

Item	Sobs	Shannon	Simpson	Ace	Chao	Coverage
CON	78.0	1.90	0.26	90.0	88.8	1.00
FRU	79.0	1.83	0.26	91.7	88.7	1.00
PEC	76.3	1.99	0.22	91.7	91.0	1.00
STA	79.7	1.96	0.23	96.5	99.8	1.00
*P*-value	NS^a^	NS	NS	NS	NS	NS

*^a^NS, not significant.*

### Silage Bacteria Changes

In total, eight bacterial phyla, 11 classes, 24 orders, 41 families, 57 genera, and 81 species were identified in the silage samples. Among these phyla, *Firmicutes* had relatively high abundances, with a mean abundance of 99.09% (Supplementary Figure S3). Among the families, *Leuconostocaceae* (50.66%) and *Lactobacillaceae* (48.35%) had relatively high abundances (Supplementary Figure S3).

**FIGURE 2:**
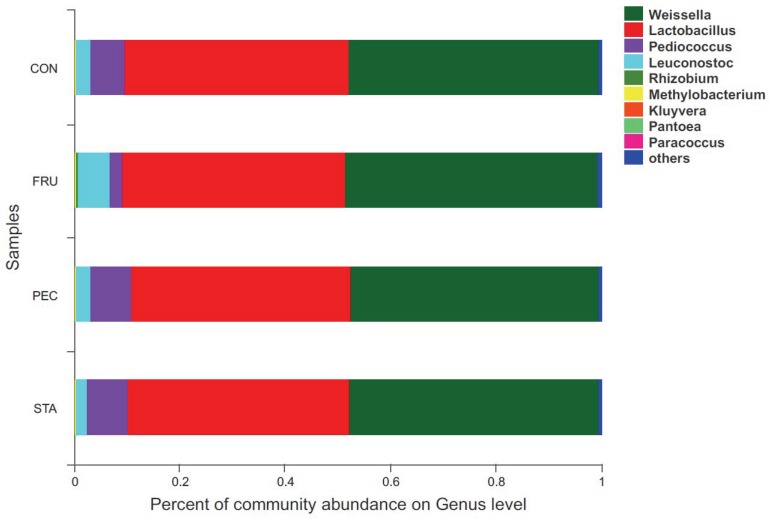
Relative abundance of bacteria community proportions at the genus level across the treatments with different sugar sources (as a percentage of the total sequence).

**FIGURE 3:**
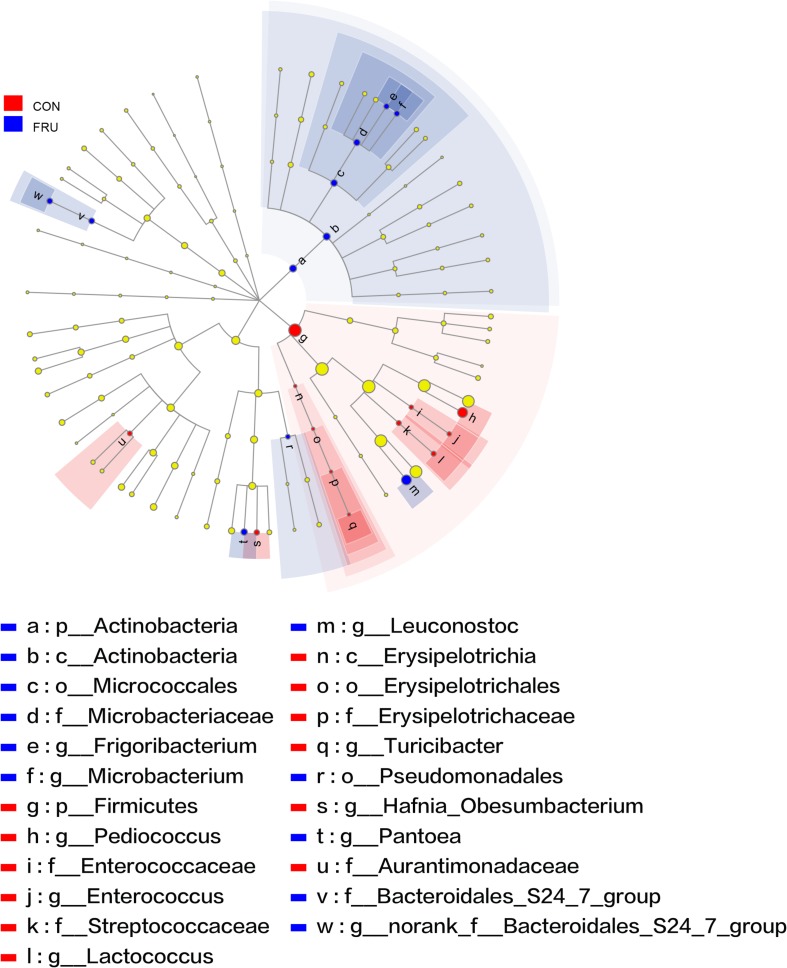
The bacteria (highlighted by small circles and by shading) with different abundances between the fructose (FRU) and control (CON) groups. There are six layers from the inside of this plot to the outside, corresponding to six levels of taxonomy (kingdom, phylum, class, order, family, and genus). Each node (small circle) represents a taxon. Blue and red nodes represent the bacterial communities with significantly higher and lower abundances in FRU compared to CON, respectively. Yellow nodes indicate the bacteria that were not statistically or biologically different between the two groups. The diameter of each circle is proportional to the abundance of the taxon.

**FIGURE 4:**
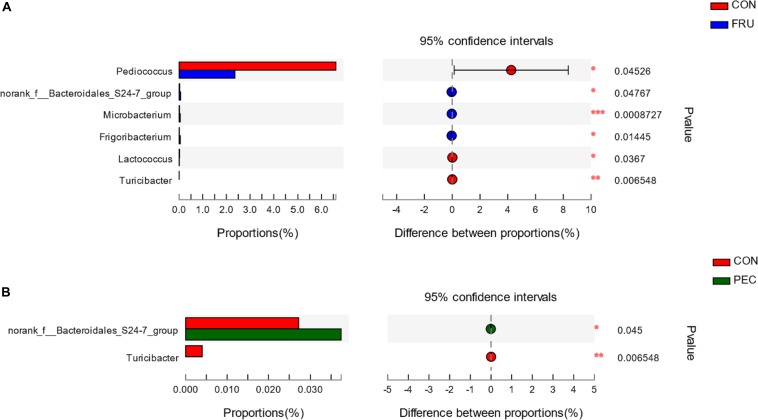
The bacterial community at the genus level was significantly different between the CON group and FRU group **(A)** and between the CON group and PEC group **(B)**. Data are shown as relative abundance (%) of each genus in each group. Statistical analysis was performed with the Mann–Whitney *U*-test. *n* = 3, in each group. *P < 0.05, **P < 0.01, ***P < 0.001.

There were 57 bacterial taxa identified at the genus level, and 51 genera were present in all samples (Figure 2). *Weissella* (47.44%), *Lactobacillus* (42.13%), *Pediococcus* (6.23%), *Treponema_2* (3.22%), and *Leuconostoc* (3.22%) were considered high-abundance taxa. LEfSe identified 23 discriminative features (LDA score ≥ 2.5) whose relative abundance varied significantly between FRU and CON (Figure 3). At the phylum level, *Firmicutes* was increased, and *Actinobacteria* was decreased in FRU compared to CON. At the genus level, the abundance of *Leuconostoc*, *Microbacterium*, *norank_f__Bacteroidales_S24_7_group*, *Frigoribacterium*, and *Pantoea* was greater, and the abundance of *Turicibacter*, *Enterococcus*, *Hafnia_Obesumbacterium*, *Lactococcus*, and *Pediococcus* was lower in FRU than in CON. No discriminative features were detected as discriminative features (LDA score ≥ 2.5) between PEC and CON or between STA and CON. Using the Mann–Whitney *U*-test, at the genus level, the abundance of *Pediococcus*, *Lactococcus*, and *Turicibacter* was greater in CON than in FRU, but the abundance of *norank_f__Bacteroidales_S24_7_group*, *Microbacterium*, and *Frigoribacterium* was greater in FRU compared to CON. The abundance of *norank_f__Bacteroidales_S24-7_group* was higher in PEC than in CON, but the abundance of *Turicibacter* was greater in CON than in PEC (Figure 4). No discriminative features were detected between STA and CON.

### Identification of Metabolites

Both positive and negative models contained 24 samples and four QC samples. Among these, 780 peaks with metabolite names were positive, and 815 peaks with metabolite names were negative. In the positive model, 32, 20, and three peaks were identified as DMs between FRU and CON, PEC and CON, STA and CON, respectively. In the negative model, 12, 17, and one peaks were identified as DMs between FRU and CON, PEC and CON, STA and CON, respectively.

The detailed parameters for the DMs are listed in Supplementary Table S4. In total, 35 DMs were upregulated, and nine DMs were downregulated in FRU compared to CON. There were 25 DMs that were upregulated, but 12 DMs were downregulated in PEC compared to CON. There was one DM that was upregulated and three DMs that were downregulated in STA compared to CON. A total of 23 mutual metabolites were identified between the DMs in FRU vs. CON and PEC vs. CON, two mutual metabolites were identified between the DMs in PEC vs. CON and STA vs. CON, 21 metabolites were only found in FRU vs. CON, 12 metabolites were only found in PEC vs. CON, and two metabolites were only found in STA vs. CON. After 60 days of ensiling, samples treated with FRU and PEC presented more peptides, such as Leu–Val–Thr, Leu–Phe, Ile–Pro–Ile, Val–Trp, and Ile–Leu–Leu, than the CON samples, but a lower abundance of metabolites for triterpene glycosides such as sanchinoside B1, medicagenic acid, betavulgaroside IV, and prosapogenin compared to CON. The addition of PEC presented more phenyllactic acid compared to CON.

### Correlation Between the Microbiome and Fermentation Characteristics

**FIGURE 5:**
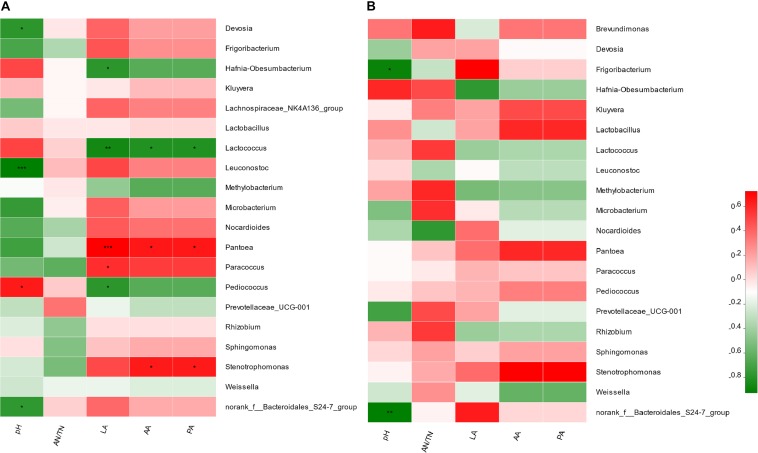
Correlation matrix between the fermentation and nutritional characteristics and the top 20 enriched bacteria at the genus level from the comparisons between the CON group and FRU group **(A)** and between the CON group and PEC group **(B)**. Positive correlations are shown in red, and negative correlations are shown in green. Color intensity is proportional to the correlation values (*r* ≥ 0.59) within a correlation group. *mean *P* < 0.05, **mean *P* < 0.01, ***P < 0.001.

In the comparison between FRU and CON, it was found that *Pediococcus* (*r* = 0.89, *P* < 0.05) was positively correlated with the pH value, and *Leuconostoc* (*r* = −0.99, *P* < 0.001) and *norank_f__Bacteroidales_S24-7_group* (*r* = −0.81, *P* < 0.05) were negatively correlated with the pH value (Figure 5A). *Pantoea* (*r* = 0.99, *P* < 0.001) was positively correlated with lactic acid, and *Pediococcus* (*r* = −0.83, *P* < 0.05) and *Lactococcus* (*r* = −0.93, *P* < 0.01) were negatively correlated with lactic acid. *Pantoea* was positively correlated with acetic acid and propionic acid, and *Lactococcus* was negatively correlated with acetic acid and propionic acid. In the comparison between PEC and CON, the *norank_f__Bacteroidales_S24-7_group* (*r* = –0.93, *P* < 0.05) was negatively correlated with the pH value (Figure 5B).

### Linkages Between Microbial and Metabolomic Profiles

**FIGURE 6:**
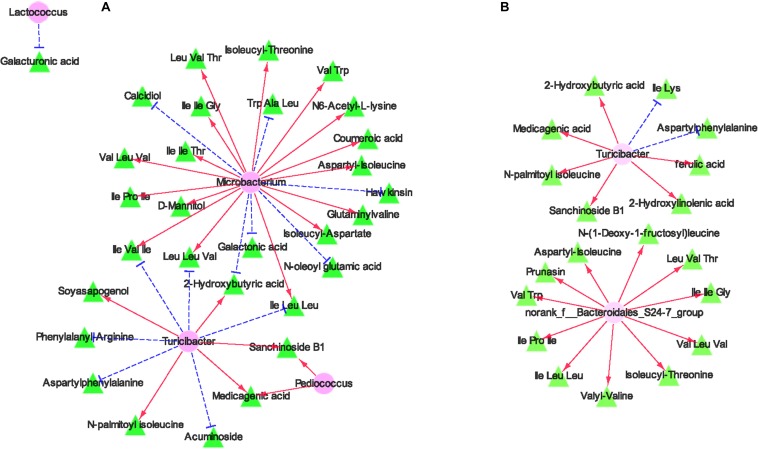
A co-occurrence network analysis among the different microbiota and metabolites from the comparisons between the CON group and FRU group **(A)** and between the CON group and PEC group **(B)**. Each co-occurring pair among microbial populations at the genus level and metabolites has an absolute Spearman rank correlation above 0.94 [red straight line, positive correlation (*r* ≥ 0.94); blue dotted line, negative correlation (*r* ≤ -0.94)]. Microbes are shown by green V-shaped nodes, and metabolites are shown by pink round nodes.

In the comparison between FRU and CON (Figure 6A), *Microbacterium* was positively correlated with Ile–Ile–Thr, Leu–Val–Thr, Ile–Ile–Gly, D-mannitol, Val–Leu–Val, isoleucyl–threonine, glutaminylvaline, coumaric acid, Leu–Leu–Val, Ile–Pro–Ile, Val–Trp, Ile–Leu–Leu, isoleucyl-aspartate, N6-acetyl-L-lysine, aspartyl-isoleucine, and Ile–Val–Ile and negatively correlated with calcidiol, galactonic acid, Trp–Ala–Leu, N-oleoyl glutamic acid, hawkinsin, and 2-hydroxybutyric acid. *Pediococcus* was positively correlated with sanchinoside B1 and medicagenic acid. *Turicibacter* was positively correlated with sanchinoside B1, soyasapogenol, *N*-palmitoyl isoleucine, 2-hydroxybutyric acid, and medicagenic acid and negatively correlated with Leu–Leu–Val, Ile–Leu–Leu, acuminoside, Ile–Val–Ile, phenylalanyl-arginine, and aspartylphenylalanine. *Lactococcus* was negatively correlated with galacturonic acid. In the comparison between PEC and CON (Figure 6B), *norank_f__Bacteroidales_S24-7_group* was positively correlated with *N*-(1-deoxy-1-fructosyl)leucine, Leu–Val–Thr, Ile–Ile–Gly, Val–Leu–Val, isoleucyl-threonine, valyl-valine, Ile–Leu–Leu, Ile–Pro–Ile, Val–Trp, prunasin, and aspartyl-isoleucine. *Turicibacter* was positively correlated with ferulic acid, 2-hydroxylinolenic acid, sanchinoside B1, *N*-palmitoyl isoleucine, medicagenic acid, and 2-hydroxybutyric acid and negatively correlated with Ile–Lys and aspartylphenylalanine.

## Discussion

Relatively high-quality silage should have a pH value of less than 4.2, a content lower than 100 g kg^–1^ total N, and a butyric acid content of less than 10 g kg^–1^ dry matter (McDonald et al., 1991). Thus, the results from the current experiment demonstrated that FRU and PEC silage were well preserved with relatively lower pH values and higher lactic acid contents, but this was not observed for STA and CON. The changes in the pH value and lactic acid concentration in the current study might be due to a different microbial community and metabolic process present under the various sugar sources.

Considering the bacterial community, the main silage bacterial phylum in the current study was *Firmicutes*, which was the same as the results of a previous study that detected a relative abundance of 95% *Firmicutes* in alfalfa silage after 60 days (McGarvey et al., 2013). McGarvey et al. (2013) reported that *Lactobacillus* was the most abundant out of the 10 most commonly isolated OTUs from alfalfa silage. However, the highly abundant genera in the current study, *Lactobacillus*, *Enterococcus*, *Streptococcus*, and *Weissella*, were different from the observed microbiota in previous studies of natural alfalfa or alfalfa silage (Guo et al., 2018); however, the two dominant genera identified in this study, *Lactobacillus* and *Weissella*, were similar to our latest research (Zhang et al., 2018). It was found that the dominant genera *Weissella* and *L. plantarum* were found in corn, sorghum, and forage paddy rice silages, but *Leuconostoc* were the dominant species in alfalfa silage (Pang et al., 2011). Thus, the dominant three genera, *Weissella*, *Lactobacillus*, and *Leuconostoc*, might be good indicators for alfalfa ensiling. The genus *Weissella* is assigned to the phylum *Firmicutes*, class *Bacilli*, order *Lactobacillales*, and family *Leuconostocaceae* (Collins et al., 1993). In addition to *Weissella* and *Lactobacillus*, the bacteria in alfalfa silage belong to an additional 51 genera, some of which were reported for the first time in silage such as *Kluyvera* and *Pantoea*. Therefore, Illumina MiSeq high-throughput sequencing is an effective platform to obtain additional novel information to survey bacterial community composition from silage samples.

It was reported that true proteins can be degraded into peptides, free amino acids, and AN due to proteolysis during the ensiling of *M. sativa* (McDonald et al., 1991). The supplementation of sugars would generate lactic acid more quickly by WSC and soluble nitrogen sources during the initial fermentation period (Muck et al., 2018). The quick decrease in pH value by the addition of WSC would inhibit the enzymatic activity of ensiling bacteria and reduce the ability of the bacteria to utilize the peptides (Guo et al., 2007), which might have resulted in the relatively greater number of small peptides in FRU and PEC compared to CON. In a previous study, a greater abundance of threonine, tyrosine, valine, lysine, aspartic acid, and alanine was associated with better ensiling quality in alfalfa silage supplemented with *L. plantarum* compared to CON, which might indicate that the high-quality alfalfa silage generated by additives could protect the degradation of true protein sources to AN (Guo et al., 2018). However, rare small peptides were detected by GC-MS (Guo et al., 2018). The analytes detected by GC-MS require volatile procedures and laborious derivatization (Lee et al., 2013). Thus, the category of metabolites different from previous studies might be mainly due to analytical technology. The current study is the first to use LC-MS metabolomics to detect the metabolome of alfalfa silage, which has the advantage of detecting the large molecular weight metabolites and better properties in preserving the structural integrity of thermolabile analytes than GC-MS (Lee et al., 2013).

Betavulgaroside IV is a representative triterpene seco-glycoside that constitutes a small family of plant saponins (Zhu et al., 2008). It was reported that alfalfa saponins are pentacyclic triterpenes, including medicagenic acid, soyasapogenol, and other glycosides of several aglycones (Szumacher-Strabel et al., 2019). The total saponin concentration increases when fresh alfalfa is processed into silage (Szumacher-Strabel et al., 2019). An apparent negative linear relationship was observed between protein and saponin content in alfalfa silage (Szumacher-Strabel et al., 2019). Thus, the decreased level of triterpene aglycones in FRU and PEC compared to CON also indicated the improved fermentation quality of alfalfa silage after adding fructose or pectin. In addition, saponins have antimicrobial abilities (Wang et al., 2012), inhibiting feed intake, rumen fermentation, nutrient digestibility, and animal performance at a relatively high dosage (Klita et al., 1996; Wang et al., 2017). Thus, the decreased level of triterpenes in FRU and PEC compared to CON might indicate the improved feed quality of alfalfa silage treated with FRU or PEC when used for ruminants. Moreover, sanchinoside B1, prosapogenin, betavulgaroside IV, tragopogonsaponin J, and myricatomentoside II have not been previously identified as alfalfa saponins in alfalfa silage. Alfalfa is also an important dietary ingredient as a source of vitamins for dairy cows (Hojilla-Evangelista et al., 2017). *Lactobacillus* strains need vitamins (Jaramillo et al., 2018) due to their limited ability to synthesize B vitamins (Gao et al., 2011). In the current study, we found that calcidiol, the metabolite of vitamin D3, was lower in FRU, but biocytin and acuminoside were greater in FRU and PEC compared to CON, indicating the protective roles of FRU and PEC in preventing the degradation of vitamins, as the ensiling procedure easily results in the loss of vitamins (Tagawa et al., 2014).

The PEC silage had a much greater abundance of D-galacturonic acid compared to CON, which confirmed the utilization of supplemented pectin during LAB fermentation of alfalfa silage. Galactonic acid is the metabolite of D-galacturonic acid in the biocatalysis of D-galacturonate reductase. Thus, the increased concentration of D-galacturonic acid but decreased value of galactonic acid might be interpreted by the inhibition of D-galacturonate reductase at lower pH values (Guo et al., 2007) in PEC than in CON. Pectin that consists of D-galacturonic acid is an important constituent of alfalfa, which composes at most 10.5–14.2% of the DM basis (Mertens, 2002), but it may not be easily used under general LAB fermentation because it constitutes the plant cell wall with hemicellulose and cellulose by their interactions and ester cross-links (Broxterman and Schols, 2018). Thus, we are planning future experiments involving the addition of pectinase or pectin-degrading LAB to release pectin from plant cell walls during ensiling.

*Leuconostoc* is the major bacterial genus present from the initial to the middle stages of fermentation (Plengvidhya et al., 2007), predominantly converting pyruvate to D-lactate using D-lactate dehydrogenase with D-fructose as a carbon source. It was found that all 12 *Leuconostoc* haplotypes could utilize D-fructose (Strausbaugh, 2016). Thus, the increase abundance of *Leuconostoc* in FRU group silage might be due to the supplementation of fructose. *Pediococcus* was positively correlated with pH and sanchinoside B1 (triterpene aglycones) in the comparison between FRU and CON. Until now, however, no studies have shown the relationship between *Pediococcus* and saponin or triterpene aglycones. Dutkiewicz et al. (2016) recently demonstrated that *Pantoea* could inhibit the growth of *Escherichia coli* and fungi. Jacxsens et al. (2003) reported that *Pantoea agglomerans* can produce acetic acid, propionic acid, and succinate by fermenting sugars under anaerobic conditions. We estimated that the ability of fructose to enhance ensiling quality might be through the inhibiting roles of *Pediococcus* and accelerating roles of *Leuconostoc* and *Pantoea*, which should be verified later.

It was reported that phenyllactic acid (Ström et al., 2002; Ndagano et al., 2011), cyclic dipeptides (Ström et al., 2002), and peptides (Atanassova et al., 2003) produced by LAB had antifungal activity; there, compounds could effectively inhibit the growth of pathogens, promote the fermentation of food, and extend the shelf-life of products. The DMs of small peptides were positively correlated with *Microbacterium* in the FRU group *and norank_f__Bacteroidales_S24-7_group* in PEC, and sanchinoside B1 and medicagenic acid were positively correlated with *Pediococcus* in FRU, indicating that these metabolites and microbes are potential indicators of good quality alfalfa silage. Members of the genus *Microbacterium* have previously been isolated from a wide range of environments, such as soils and air, and from plants (Yoon et al., 2009; Alves et al., 2014). It was reported that a β-fructofuranosidase produced by *Microbacterium* sp. H-1 has strong trans-β-fructofuranosylation activity with sucrose (Ishikawa et al., 1991). A previous study also found that *Microbacterium foliorumasa* can use fructose to generate D-allulose (An et al., 2019). Thus, the increased abundance of *Microbacterium* in FRU silage compared to CON might be attributed to the presence of *Microbacterium*.

Both fructose and pectin improved the alfalfa silage quality probably through increased *norank_f__Bacteroidales_S24-7_group* and decreased *Turicibacter*. However, the FRU- and PEC-associated *norank_f__Bacteroidales_S24-7_group* and *Turicibacter* from the current study have not been reported before for their ability to utilize these sugars or be inhibited by these sugars. Thus, future studies should be included to determine their mechanism in an anaerobic environment with fructose and pectin as substrates.

## Conclusion

The addition of PEC and FRU apparently improved the fermentation quality by shifting the bacterial community and metabolomics profiles of the alfalfa silage. The addition of FRU effectively improved the relative abundance of *Leuconostoc*, *Pantoea*, and *Microbacterium* and decreased the *Pediococcus*. The addition of PEC improved the relative abundance of *norank_f__Bacteroidales_S24-7_group* and decreased the *Turicibacter*. The FRU and PEC silages presented a greater level of small peptides and a lower concentration of triterpene glycosides compared to CON silage. These bacteria and metabolites were important factors in the fermentation of alfalfa silage. The different bacteria provided new information on screening targeted functional LAB for modulating silage quality.

## Data Availability Statement

The datasets generated for this study can be found in the SRP197138.

## Author Contributions

BW and ZY conceived and designed the experiments. BW and RG conducted the experiments. BW, RG, and ZW performed the statistical analysis of the experimental data. Finally, the manuscript was written by BW and was modified by YZ. All authors read and approved the final manuscript.

## Conflict of Interest

The authors declare that the research was conducted in the absence of any commercial or financial relationships that could be construed as a potential conflict of interest.

## Footnotes

1 http://www.ncbi.nlm.nih.gov/Traces/sra/

2 http://drive5.com/uparse/

3 http://rdp.cme.msu.edu/

4 https://www.arb-silva.de/

5 http://www.genome.jp/kegg/

6 http://www.hmdb.ca/

7 http://www.metaboanalyst.ca/

8 www.i-sanger.com
