# Supplementary material for: Functional Analysis of Sugars in Modulating Bacterial Communities and Metabolomics Profiles of Medicago sativa Silage
Source: Front Microbiol. 2020 May 5;11:641. doi: 10.3389/fmicb.2020.00641 (PMC7232540; doi:10.3389/fmicb.2020.00641)
Supplement: Supplementary file 1 [file Data_Sheet_1.docx]

***Supplementary Material***

**Functional analysis of Sugars in Modulating Bacterial Communities and Metabolomics profiles of *Medicago sativa* Silage**

**Bing Wang^1^, Run Gao^2^, Zhe Wu^2^, Zhu Yu^2*^**

^1^State Key Laboratory of Animal Nutrition, College of Animal Science and Technology, China Agricultural University, Beijing 100193, P. R. China

^2^College of Grass Science and Technology, China Agricultural University, Beijing 100193, P. R. China

Running title: Alfalfa silage microbial and metabolic profiles

**^*^Correspondence:**

Dr. Zhu Yu

[yuzhu33150@sina.com](mailto:yuzhu33150@sina.com)

**Table S1.** Chemical compositions and fermentation characteristics of alfalfa before ensiling

| Items | *Medicago sativa.* |
| --- | --- |
| Dry matter (g/kg DM) | 320.3 |
| Crude protein (g/kg DM) | 194.4 |
| Neutral detergent fiber (g/kg DM) | 377.8 |
| Acid detergent fiber (g/kg DM) | 285.2 |
| pH | 6.02 |
| Ammonia nitrogen/Total nitrogen | 0.11 |
| Lactic acid (g/kg DM) | 17.4 |
| Acetate (g/kg DM) | 12.8 |
| Propionate (g/kg DM) | NF |

**Table S2.** Summary of the principal coordinate analysis (PCoA) plot showing variation in bacterial community structure (PERMANOVA test with 999 permutations)

| Characteristics | SumsOfSqs | MeanSqs | F.Model | R2 | P-value | P-adjust |
| --- | --- | --- | --- | --- | --- | --- |
| Groups | 0.1351 | 0.04503 | 2.4709 | 0.48095 | 0.032 | 0.032 |

**Table S3.** Summary of the model fitness of UHPLC-QTOF/MS analysis about the comparison between control (CON) and other three treatments (FRU, PEC, and STA).

| Model | A | R**^2^X** | R**^2^X(cum)** | R**^2^Y** | R**^2^Y(cum)** | Q**^2^** | Q**^2^(cum)** |
| --- | --- | --- | --- | --- | --- | --- | --- |
| FRU-CON |  |  |  |  |  |  |  |
| PLS-DA | p1 | 0.409 | 0.409 | 0.993 | 0.993 | 0.935 | 0.935 |
|  | p2 | 0.162 | 0.571 | 0.0046 | 0.998 | 0.519 | 0.969 |
| OPLS-DA | p1 | 0.425 | 0.425 | 0.963 | 0.963 | 0.945 | 0.945 |
|  | o1 | 0.236 | 0.661 | 0.0282 | 0.0282 | 0.0324 | 0.0324 |
|  | sum |  | 0.661 |  | 0.992 |  | 0.977 |
| PEC-CON |  |  |  |  |  |  |  |
| PLS-DA | p1 | 0.443 | 0.443 | 0.963 | 0.963 | 0.945 | 0.945 |
|  | p2 | 0.218 | 0.661 | 0.0282 | 0.992 | 0.644 | 0.98 |
| OPLS-DA | p1 | 0.425 | 0.425 | 0.963 | 0.963 | 0.945 | 0.945 |
|  | o1 | 0.236 | 0.661 | 0.0282 | 0.0282 | 0.0324 | 0.0324 |
|  | sum |  | 0.661 |  | 0.992 |  | 0.977 |
| STA-CON |  |  |  |  |  |  |  |
| PLS-DA | p1 | 0.212 | 0.212 | 0.981 | 0.981 | 0.739 | 0.739 |
|  | p2 | 0.252 | 0.464 | 0.0139 | 0.995 | 0.448 | 0.856 |
| OPLS-DA | p1 | 0.207 | 0.207 | 0.981 | 0.981 | 0.739 | 0.739 |
|  | o1 | 0.258 | 0.464 | 0.0139 | 0.0139 | 0.126 | 0.126 |
|  | sum |  | 0.464 |  | 0.995 |  | 0.865 |

**Table S4.** Candidate ruminal metabolites that differed between the control (CON) and fructose (FRU), pectin (PEC), and starch (STA) treatment.

| Metabolite | Mode | VIP | FDR | Log_2_FC |
| --- | --- | --- | --- | --- |
| *FRU vs. CON* |  |  |  |  |
| Val Arg | pos | 1.28 | 8.0E-07 | 6.61 |
| Ile Ile Thr | pos | 1.30 | 7.5E-07 | 6.23 |
| Leu Val Thr | pos | 2.34 | 1.6E-09 | 4.41 |
| Ile Ile Gly | pos | 1.94 | 1.2E-08 | 3.43 |
| D-Mannitol | neg | 5.82 | 4.2E-08 | 3.41 |
| Val Leu Val | pos | 1.46 | 3.4E-08 | 3.12 |
| Isoleucyl-Threonine | pos | 2.08 | 3.1E-08 | 3.10 |
| Glutaminylvaline | pos | 2.55 | 2.8E-08 | 3.06 |
| Coumeroic acid | pos | 1.50 | 2.9E-08 | 3.01 |
| Leu Leu Val | pos | 1.34 | 5.0E-08 | 2.48 |
| Ile Lys | pos | 1.70 | 1.6E-06 | 2.23 |
| Ile Pro Ile | pos | 2.69 | 3.3E-08 | 2.08 |
| Val Trp | pos | 4.71 | 5.3E-09 | 1.98 |
| Ile Leu Leu | pos | 1.95 | 3.1E-08 | 1.95 |
| Isoleucyl-Aspartate | neg | 1.62 | 6.1E-09 | 1.94 |
| Val Pro Val | pos | 1.27 | 4.6E-06 | 1.61 |
| N6-Acetyl-L-lysine | neg | 1.61 | 4.4E-08 | 1.55 |
| Trp Leu | pos | 1.14 | 7.6E-08 | 1.50 |
| Prunasin | neg | 1.62 | 7.0E-05 | 1.35 |
| Leu Phe | pos | 4.10 | 9.7E-09 | 1.35 |
| N-Malonyltryptophan | neg | 4.36 | 6.2E-07 | 1.34 |
| Aspartyl-Isoleucine | neg | 1.91 | 8.4E-08 | 1.19 |
| Acuminoside | neg | 3.65 | 3.1E-07 | 1.17 |
| Galacturonic acid | neg | 1.21 | 2.5E-06 | 1.17 |
| Biocytin | pos | 3.28 | 5.1E-07 | 1.13 |
| Ile Val Ile | pos | 1.25 | 1.1E-06 | 1.11 |
| Phenylalanyl-Arginine | pos | 1.31 | 1.4E-07 | 1.08 |
| Glutamylproline | pos | 1.82 | 1.7E-07 | 1.03 |
| Trp Pro | pos | 1.13 | 9.7E-05 | 1.01 |
| Aspartylphenylalanine | pos | 1.45 | 6.6E-06 | 0.99 |
| Isoleucylproline | pos | 3.16 | 1.7E-05 | 0.81 |
| Sanchinoside B1 | pos | 1.16 | 1.1E-03 | -0.83 |
| Calcidiol | pos | 1.32 | 6.9E-09 | -0.86 |
| Soyasapogenol | pos | 3.02 | 1.0E-04 | -0.89 |
| Betavulgaroside IV | neg | 3.00 | 5.3E-08 | -0.94 |
| Galactonic acid | neg | 1.55 | 1.0E-08 | -1.09 |
| Prosapogenin | pos | 2.60 | 7.7E-06 | -1.19 |
| Trp Ala Leu | pos | 1.15 | 3.1E-05 | -1.39 |
| N-oleoyl glutamic acid | pos | 1.31 | 1.1E-06 | -1.58 |
| Medicagenic acid | neg | 1.41 | 6.5E-05 | -1.80 |
| N-palmitoyl isoleucine | pos | 1.34 | 5.4E-06 | -2.16 |
| Thr Glu Ile Pro | pos | 1.41 | 6.9E-04 | -2.35 |
| Hawkinsin | pos | 1.78 | 1.4E-07 | -2.45 |
| 2-Hydroxybutyric acid | neg | 2.75 | 1.4E-09 | -3.72 |
| *PEC vs. CON* |  |  |  |  |
| Leu Val Thr | pos | 1.70 | 1.4E-06 | 3.85 |
| Ile Ile Gly | pos | 1.50 | 9.4E-08 | 3.05 |
| Val Leu Val | pos | 1.24 | 1.3E-06 | 3.00 |
| Isoleucyl-Threonine | pos | 1.53 | 1.7E-07 | 2.61 |
| Valyl-Valine | pos | 2.34 | 2.6E-07 | 1.96 |
| Ile Leu Leu | pos | 1.71 | 1.6E-06 | 1.90 |
| N-(1-Deoxy-1-fructosyl)leucine | pos | 1.12 | 8.5E-06 | 1.82 |
| Ile Lys | pos | 1.22 | 1.1E-07 | 1.74 |
| Ile Pro Ile | pos | 2.04 | 2.4E-07 | 1.73 |
| Acuminoside | neg | 5.05 | 8.4E-09 | 1.70 |
| Val Trp | pos | 3.41 | 1.5E-06 | 1.56 |
| Tragopogonsaponin J | neg | 1.45 | 1.3E-05 | 1.51 |
| N-Malonyltryptophan | neg | 4.49 | 4.1E-07 | 1.33 |
| Prunasin | neg | 1.54 | 1.9E-06 | 1.17 |
| Myricatomentoside II | neg | 2.16 | 3.9E-07 | 1.16 |
| Biocytin | pos | 2.99 | 2.6E-07 | 1.14 |
| N6-Acetyl-L-lysine | neg | 1.26 | 3.2E-10 | 1.08 |
| Lysinoalanine | neg | 1.77 | 1.3E-04 | 1.03 |
| D-Galacturonic acid | neg | 1.13 | 4.5E-07 | 1.01 |
| Leu Phe | pos | 2.95 | 6.2E-07 | 1.01 |
| Aspartyl-Isoleucine | neg | 1.70 | 3.7E-08 | 0.97 |
| L-Agaritine | neg | 1.14 | 5.3E-05 | 0.95 |
| Indolelactic acid | neg | 2.39 | 7.9E-07 | 0.90 |
| Phenyllactic acid | neg | 9.60 | 2.3E-08 | 0.81 |
| Aspartylphenylalanine | pos | 1.14 | 3.6E-05 | 0.81 |
| ferulic acid | neg | 1.21 | 1.7E-04 | -0.85 |
| Glycyrrhetinic acid | pos | 1.40 | 6.2E-06 | -0.94 |
| Betavulgaroside IV | neg | 3.23 | 2.3E-07 | -1.08 |
| Hawkinsin | pos | 1.26 | 3.2E-06 | -1.09 |
| 2-Hydroxylinolenic acid | neg | 2.06 | 3.6E-06 | -1.16 |
| Sanchinoside B1 | pos | 1.26 | 1.6E-04 | -1.19 |
| Prosapogenin | pos | 2.43 | 4.5E-06 | -1.36 |
| Glucosylsphingosine | pos | 2.26 | 5.1E-07 | -1.44 |
| Lysyl-Valine | pos | 3.79 | 5.1E-07 | -1.48 |
| N-palmitoyl isoleucine | pos | 1.18 | 1.2E-05 | -1.88 |
| Medicagenic acid | neg | 1.52 | 3.7E-05 | -2.04 |
| 2-Hydroxybutyric acid | neg | 2.71 | 3.7E-09 | -2.77 |
| *STA vs. CON* |  |  |  |  |
| Octadecanedioic acid | neg | 1.74 | 1.5E-06 | 6.25 |
| Glucosylsphingosine | pos | 4.05 | 1.5E-07 | -1.50 |
| Lysyl-Valine | pos | 6.75 | 2.6E-07 | -1.53 |
| Spirolide E | pos | 6.44 | 3.9E-07 | -1.90 |


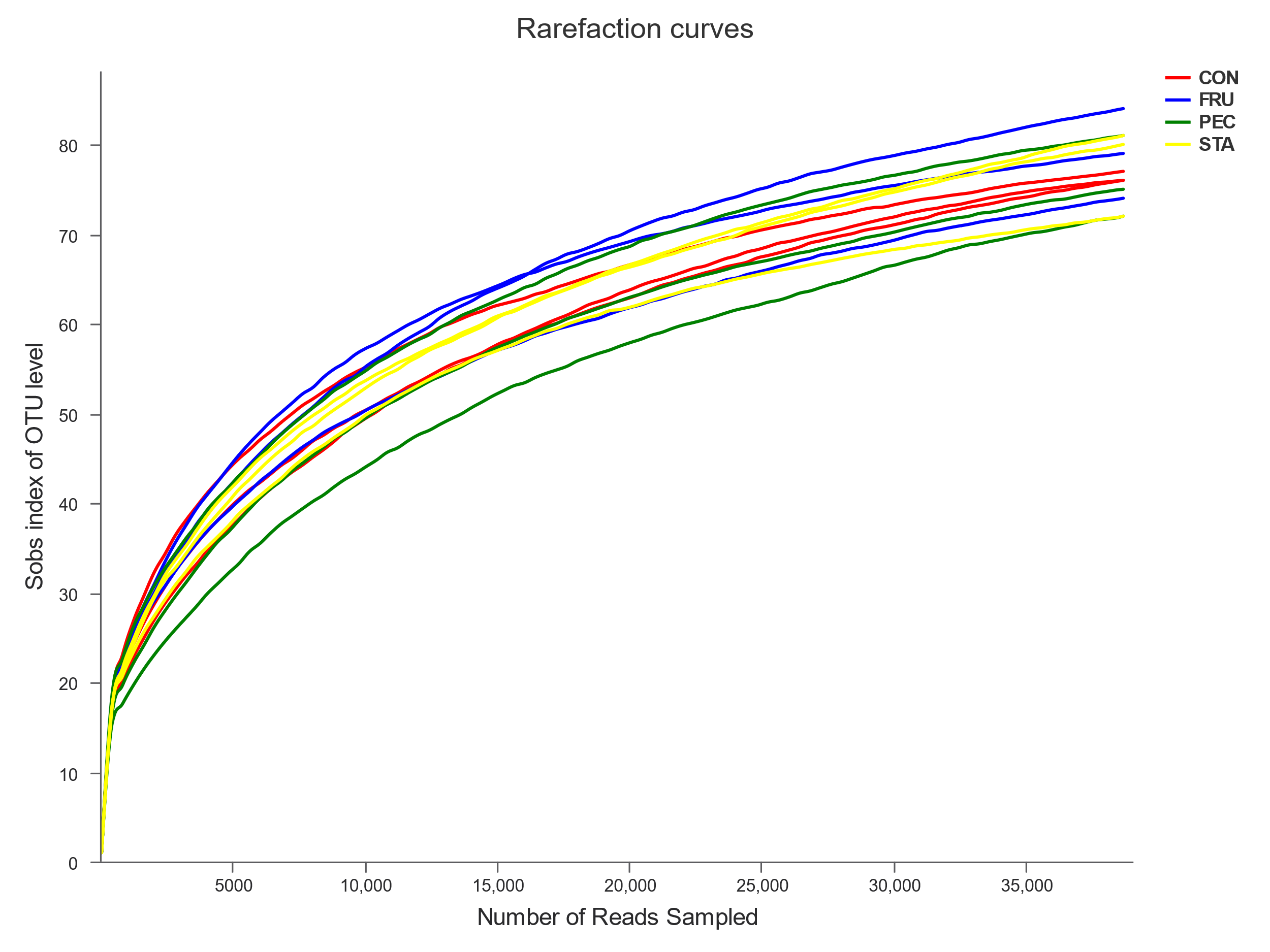


**Figure S1.** The rarefaction curves of the 16S rRNA gene reads derived from the Sobs index of OUT level after normalization.

**Figure S2.** Venn diagram illustrating overlap of microbial operational taxonomic units (OTUs) at 3% dissimilarity level among treatments.


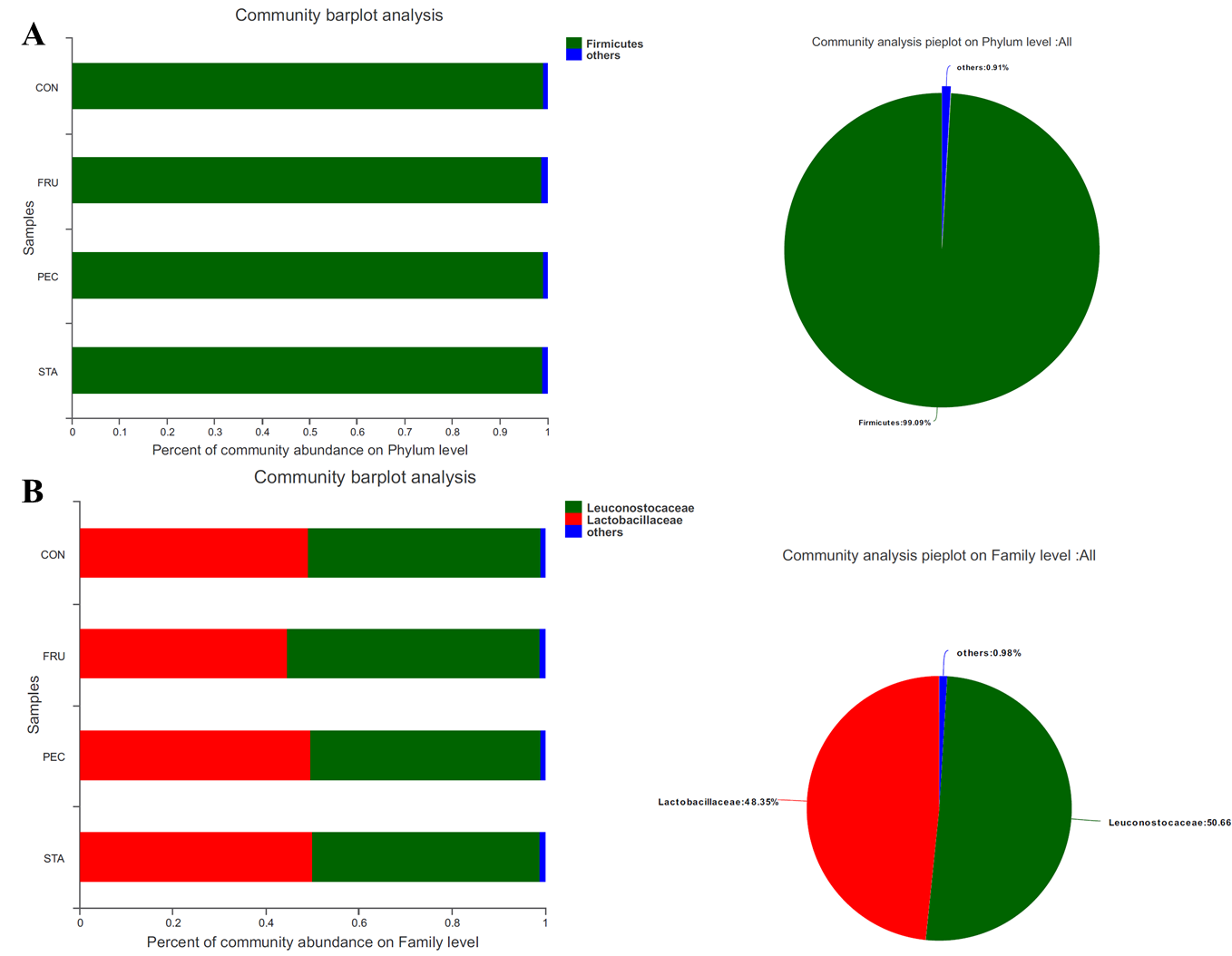


**Figure S3.** Distribution of bacterial taxonomy including phylum (A) and family (B) for the percentage of community abundance.
